# Whole genome sequencing reveals virulence–mobile element linkages and phylogenetic diversity in multidrug-resistant *Escherichia coli* from Nigeria

**DOI:** 10.3389/fmicb.2025.1579175

**Published:** 2025-05-07

**Authors:** Nubwa Medugu, Mabel Kamweli Aworh, Kenneth Iregbu, Philip Nwajiobi-Princewill, Dawn M. Hull, Lyndy Harden, Pallavi Singh, Stephen Obaro, Abiodun Egwuenu, Faith Adeboye, Ruth Egah, Leonard Uzairue, Yahaya Mohammed, Nwafia Ifeyinwa, Siddhartha Thakur

**Affiliations:** ^1^Department of Medical Microbiology and Immunology, Nile University of Nigeria, Abuja, Nigeria; ^2^Department of Medical Microbiology and Parasitology, National Hospital Abuja, Abuja, Nigeria; ^3^Department of Biological and Forensic Sciences, Fayetteville State University, Fayetteville, NC, United States; ^4^Department of Population Health and Pathobiology, College of Veterinary Medicine, North Carolina State University, Raleigh, NC, United States; ^5^Department of Biological Sciences, Northwestern Illinois University, DeKalb, IL, United States; ^6^Department of Paediatric Infectious Diseases, University of Alabama at Birmingham, Birmingham, AL, United States; ^7^Nigeria Centre for Disease Control, Abuja, Nigeria; ^8^International Foundation against Infectious Disease in Nigeria, Abuja, Nigeria; ^9^Department of Medical Laboratory Science, Federal University, Oye, Nigeria; ^10^Department of Microbiology, Usmanu Danfodiyo University Sokoto, Sokoto, Nigeria; ^11^Department of Medical Microbiology, University of Nigeria, Nsukka, Nigeria

**Keywords:** multidrug-resistant *Escherichia coli*, virulence factors, phylogenetic diversity, whole-genome sequencing (WGS), mobile genetic elements (MGEs), IncFII plasmids, antimicrobial resistance (AMR), pathotypes

## Abstract

**Background:**

Multidrug-resistant *Escherichia coli* poses a critical public health threat in Nigeria, where limited genomic surveillance hinders the understanding of virulence-resistance interplay.

**Methods:**

This cross-sectional study employed whole-genome sequencing to characterize 107 MDR-E isolates from a Nigerian tertiary hospital (2019–2020), analyzing virulence genes, mobile genetic elements (MGEs), phylogroups, sequence types (STs), pathotypes, and antimicrobial resistance (AMR).

**Results:**

We identified 2,021 virulence genes across nine functional categories, dominated by immune evasion (*terC*, 96.3%), adherence (*fimH*, 86%), and iron acquisition (*fyuA*, 63.6%). Strikingly, 81.3% of virulence genes were linked to MGEs, including MITEEc1 (75.7% of isolates) and IS30 (56.1%), with IncFII (17.8%) and Col156 (12.1%) plasmids co-harboring virulence (e.g., *traJ/traT*, *senB*) and AMR genes (e.g., *blaTEM-1B*). Phylogroup B2 (32.7%) dominated, exhibiting high resistance to ampicillin (97.1%) and emerging meropenem resistance (11.4%). Globally disseminated STs (ST131, ST410, ST648) carried significantly more diverse virulence genes than minor clones (*p* = 0.028) and were strongly associated with double-serine QRDR mutations (*gyrA_S83L*: 97.6%, *parC_S80I*: 97.6%), which correlated with more virulence genes (24.2 vs. 22.3 genes) and resistance (MAR index: 0.7 vs. 0.5) compared to minor clones. Notably, 92% (61/66) of high-risk clones harbored these mutations, versus 57% (21/37) of low-risk clones, suggesting a fitness advantage enabling major clones to sustain larger genetic cargoes. Pathotyping revealed 54.2% as extraintestinal pathogenic *E. coli* (ExPEC), with 72.4% of these being uropathogenic (UPEC) and 5.2% ExPEC/EAEC hybrids, alongside 43.9% atypical ExPEC strains. Hierarchical clustering demonstrated phylogroup B2’s genetic diversity and co-localization of plasmid-borne virulence/AMR genes.

**Discussion:**

These findings underscore Nigeria’s MDR-E crisis, driven by MGE-facilitated gene transfer, hybrid pathotypes, and globally disseminated high-risk clones harboring double-serine QRDR mutations. There is continued need for robust genomic surveillance, stringent infection control measures, enhanced antibiotic stewardship, and exploration of antivirulence strategies (e.g., targeting *fimH* or *yeh*) to curb the spread of these highly adaptable pathogens in resource-limited settings and beyond.

## 1 Introduction

Multidrug-resistant *Escherichia coli* (MDR-E) poses a significant public health challenge, particularly in low- and middle-income countries (LMICs), where it contributes substantially to morbidity and mortality in severe infections such as sepsis, meningitis, and urinary tract infections (UTIs) (Aiken et al., 2023; Medugu et al., 2022). The global rise of MDR-E is exacerbated by the rapid dissemination of antimicrobial resistance (AMR) genes and virulence factors, often facilitated by mobile genetic elements (MGEs) such as plasmids, transposons, and insertion sequences (Partridge et al., 2024; Schmidt and Hensel, 2004; World Health Organization, 2022).

Pathogenic *E. coli* strains differ from commensal strains by the presence of virulence genes, which encode factors involved in adherence, colonization, invasion, and immune evasion (Nojoomi and Ghasemian, 2019). Extraintestinal pathogenic *E. coli* (ExPEC) are particularly concerning due to their extensive repertoire of virulence-associated factors, including adhesins, toxins, iron acquisition systems, and immune modulators, many of which are carried on MGEs (Nojoomi and Ghasemian, 2019; Sora et al., 2021). Despite their critical role in bacterial pathogenicity, the mechanisms by which MGEs contribute to the spread of virulence and AMR genes—such as horizontal gene transfer—remain poorly understood, particularly in resource-limited settings like Nigeria (Iheanacho and Eze, 2022; Morales et al., 2023; Partridge et al., 2024).

Phylogenetic diversity further complicates the epidemiology of *E. coli*, as different phylogroups exhibit varying degrees of virulence and AMR profiles. For instance, phylogroup B2 is frequently associated with extraintestinal infections, while other phylogroups are more commonly found in commensal gut flora (Clermont et al., 2019; Nojoomi and Ghasemian, 2019). In Nigeria, the burden of MDR-E is exacerbated by limited surveillance, inadequate antimicrobial stewardship, and widespread misuse of antibiotics, driven by factors such as limited healthcare infrastructure and over-the-counter antibiotic availability (Okeke et al., 1999). Although previous studies have characterized AMR trends in *E. coli*, there remains a gap in understanding the genomic epidemiology of virulence factors, their association with MGEs, sequence types, and pathotypes in this region. This study seeks to address this gap by leveraging whole-genome sequencing (WGS) and *in silico* analyses to characterize MDR-E isolates from Nigerian patients. Specifically, classify phylogroups, examine the distribution of virulence genes, pathotypes, and identify associated MGEs, while assessing their relationship with AMR patterns and multilocus sequence types (MLST). By providing essential insights into the genomic landscape of MDR-E in Nigeria, this study seeks to inform tailored infection prevention strategies, enhance antimicrobial stewardship programs, and ultimately improve patient outcomes in resource-limited settings.

## 2 Materials and methods

### 2.1 Study design, sample collection, transport, and initial processing

This cross-sectional study was undertaken at the National Hospital Abuja (NHA), a 425-bed tertiary care center in Nigeria. A total of 107 unique *E. coli* isolates, collected from clinical specimens between March 2019 and September 2020, were included. All specimens were obtained according to standard clinical protocols and transported promptly to the microbiology laboratory (Iregbu et al., 2013). Urine specimens were collected as midstream clean-catch. To assess for significant bacteriuria, upon receipt, urine samples were inoculated unto Brilliance UTI Agar (Oxoid, Basingstoke, United Kingdom) using a calibrated 1 μL loop. Plates were incubated at 35.5°C for 18–24 h in ambient air.

Blood specimens were collected using weight-based guidelines, with two sets of 8–10mL drawn for adult patients and age-appropriate volumes for pediatric patients. Samples were inoculated into BacT/Alert culture bottles—Adult Aerobic, Adult Anaerobic, or Pediatric Plus, as appropriate (bioMérieux, Marcy l’Etoile, France)—and incubated in the BacT/Alert automated system. Bottles were continuously monitored for microbial growth for up to 5 days, with positive signals prompting immediate subculture for organism isolation and identification. Bottles flagged as positive were subcultured onto Sheep Blood Agar, Chocolate Agar, and MacConkey Agar. Plates were incubated at 35.5°C for 18–24 h, with Chocolate Agar placed in a 5% CO2 atmosphere to support fastidious organisms. All negative bottles were incubated for the full monitoring 5-day period before being discarded as no growth (Medugu et al., 2023). Other specimen types (e.g., wound swabs, sputum, and body fluids) were inoculated on Sheep Blood Agar, Chocolate Agar, and MacConkey Agar, with incubation conditions as described above. All plates were examined after 18–24 h for colony.

### 2.2 Identification and antimicrobial susceptibility testing of *Escherichia coli* isolates

All presumptive *Escherichia coli* isolates were subjected to species-level identification using the VITEK 2 Compact system (bioMérieux, Marcy l’Etoile, France), employing the GN ID card, which contains a panel of biochemical tests specific for Gram-negative bacteria. Bacterial suspensions were prepared in sterile saline to a 0.5 McFarland turbidity standard using a Densichek turbidity meter and then loaded into the VITEK 2 instrument according to the manufacturer’s instructions. Antimicrobial susceptibility testing was performed on confirmed isolates using the VITEK 2 AST-N280 and AST-N281 cards. Quality control was conducted using *E. coli* ATCC^®^ 25922. The AST results were automatically generated by the VITEK system and interpreted using the Clinical and Laboratory Standards Institute (CLSI) guidelines, M100, 30th Edition (Clinical and Laboratory Standards Institute, 0000). Interpretations included categorization of each isolate as susceptible, intermediate, or resistant based on minimum inhibitory concentration breakpoints. All results were reviewed and validated by trained clinical microbiologists. Minimum inhibitory concentration results were validated using *E. coli* ATCC 25922 as a quality control strain. MDR was defined as resistance to three or more antimicrobial classes, consistent with established criteria (Magiorakos et al., 2012). The isolates were shipped to Thakur Molecular Epidemiology laboratory at North Carolina State University (NC State), United States where they underwent microdilution assays with the Gram-negative Sensititre (CMV3AGNF) plate (Trek Diagnostic Systems, OH). The plates were read using Sensititre ARIS and interpreted according to CLSI M100 30th Edition guidelines (Clinical and Laboratory Standards Institute, 0000). *E. coli* ATCC 25922 was used as a control. All isolates were stored at −80°C in 50% glycerol to preserve viability before further analyses. Relevant sociodemographic and clinical data, including age, sex, diagnosis, and specimen source, were extracted from patient medical records.

### 2.3 Whole-genome sequencing of *Escherichia coli* isolates, virulence gene detection, association with mobile genetic elements, and AMR gene detection in study *Escherichia coli* isolates

WGS was performed on all 107 *E. coli* isolates to characterize their genomic profiles. Genomic DNA was extracted using the MasterPure Complete DNA and RNA Purification Kit (Epicentre Technologies, Madison, WI), ensuring high-quality DNA for downstream applications. DNA quality was initially assessed using the NanoDrop 2000 spectrophotometer (Thermo Fisher Scientific, Waltham, MA, United States). Purity was evaluated by measuring the absorbance ratio at 260 and 280 nm, with a ratio of approximately 1.8 considered indicative of high-purity DNA suitable for downstream applications. Following this, DNA concentration was quantified using the Qubit 4.0 Fluorometer (Thermo Fisher Scientific) with the dsDNA High Sensitivity (HS) Assay Kit, according to the manufacturer’s instructions. Sequencing libraries were prepared using the Nextera XT DNA Sample Prep Kit (Illumina, San Diego, CA, United States). Sequencing was conducted on the Illumina MiSeq platform (Illumina, San Diego, CA, United States) with a 2 × 250 paired-end configuration, generating high-depth coverage for each isolate. Raw sequencing reads were processed using CLC Genomics Workbench v9.4 (Qiagen, Hilden, Germany) to trim low-quality bases and remove adapter sequences.

The high-quality Illumina paired-end reads generated were assembled *de novo* into the draft genome sequence for each isolate using SPAdes assembler v.3.13.1 which is optimized for bacterial genomes (Bankevich et al., 2012). The resulting draft genome sequences were subjected to rigorous quality assessment using QUAST (Quality Assessment Tool for Genome Assemblies) (Gurevich et al., 2021). Metrics such as contig number, N50, and genome coverage were evaluated to ensure the reliability and completeness of the assemblies and uploaded to NCBI’s Bio project ID PRJNA293225.

The virulence genes identified in the *E. coli* isolates were systematically categorized into nine functional categories: adherence, biofilm, and colonization; invasion and intracellular survival; toxin production and secretion; iron acquisition, uptake, transport, and storage; immune evasion and modulation; nutrient acquisition and metabolism; stress response and regulation; motility and chemotaxis; and cell wall and membrane biogenesis. To investigate the genetic context of these virulence factors, we identified MGEs associated with virulence genes using Mobile Element Finder (version 1.0.3, released on 2020-10-09) alongside its database version 1.0.2 (released on 2020-06-09) (Johansson et al., 2021). Each virulence gene was classified according to its location on plasmids, insertion sequences, other MGEs, or categorized as not associated, based on the MGE output. Plasmids were detected employing PlasmidFinder-2.0, with a threshold of at least 95% identity and a minimum coverage of 60%, utilizing draft genome assemblies (Carattoli et al., 2014). MGE detection was conducted with a threshold criterion of 95% sequence similarity and a minimum coverage of 60%, ensuring accurate identification of MGEs linked to virulence genes. Prediction of AMR was conducted by using Mobile Element Finder v1.0.3 (2020-10-09) and selecting Acquired Antimicrobial Resistance genes (ResFinder) (Johansson et al., 2021). Chromosomal point mutation data were retrieved from publicly available whole-genome sequences we had earlier deposited in the NCBI BioProject PRJNA293225 using the NCBI Pathogen Detection Isolates Browser. Mutations in the quinolone resistance-determining regions (QRDRs) of *gyrA* (Ser83) and *parC* (Ser80) were identified using the NCBI’s automated AMRFinderPlus tool (v3.10.23) with default parameters (Feldgarden et al., 2021).

### 2.4 Analysis of virulence gene carriage by sequence type (ST) and QRDR mutation prevalnece

Multilocus sequence typing (MLST) was performed using seven conserved *Escherichia coli* housekeeping genes (*adk*, *fumC*, *gyrB*, *icd*, *mdh*, *purA*, and *recA*) as previously described (Wirth et al., 2006) Sequence types (STs) and clonal complexes (CCs) were identified via the PubMLST Achtman scheme with STs assigned to isolates exhibiting 100% allele identity to established MLST databases. Isolates lacking perfect allele matches were classified as “unknown STs”(Larsen et al., 2012). The 35 identified STs were categorized as major or minor clones based on their global epidemiological prevalence and association with AMR outbreaks (Baquero et al., 2013; Manges et al., 2019; Santos et al., 2020b; Shaik et al., 2017).

To investigate the relationship between multidrug resistance and virulence profiles, we analyzed the set of *E. coli* STs represented by more than one isolate ensure robust statistical estimates, resulting in a final dataset of 20 STs. For each ST, the following variables were evaluated: (1) MAR Index (Multidrug Antibiotic Resistance Index, averaged across isolates), (2) Number of AMR genes (average count), (3) Number of virulence genes (average count), and (4) Virulence Variety (total unique virulence factors per ST). Non-parametric Spearman’s rank correlation analysis was employed to assess pairwise associations between variables, as the data exhibited non-normal distributions. Tied ranks were resolved using averaging, and significance was evaluated via a two-tailed test with an α-level of 0.05. To investigate relationship between major and minor STs with double QRDR mutations, the distinct STs classified as either major or minor clones were evaluated. Each ST was assigned to one of six categories according to the percentage of isolates exhibiting double mutations (0, 0.5, 0.75, 0.97, 0.98, and 1.0). A cross−tabulation of clone type by these double−mutation categories was constructed. Fisher’s exact test was used to assess the statistical significance of the association between clone type and double−mutation category. Statistical computations were performed in R v4.3.1.

### 2.5 Pathotyping of *Escherichia coli*

Isolates were screened for ExPEC markers according to Johnson et al. (2003). Briefly, the presence of five established virulence genes were assessed: *pap*, sfa*/foc, afa/dra, iutA*, and *kps* (Supplementary Table 6). An isolate was classified as ExPEC if two or more of these markers were present. Next, ExPEC strains were assessed using the Spurbeck/Hooton–Mobley scheme (Spurbeck et al., 2012), which categorizes isolates as UPEC if they possess *fyuA* along with at least two of the following three genes: *chuA*, *yfcV*, or *vat*. In the two schema used, each isolate was assessed for the enteroaggregative *E. coli* (EAEC) regulator gene *aggR* and when found in an isolate already tagged an ExPEC, was labeled an ExPEC/EAEC hybrid as reported previously (Lara et al., 2017). When an isolate possessed multiple ExPEC-linked genes but did not fulfill either the Johnson criteria or the Spurbeck/HM definition, it was designated “atypical ExPEC,” following the approach described by Sarowska et al. (2019). Lastly, all strains were also screened for hallmark genes of other diarrheagenic *E. coli* (DEC) pathotypes, including *eae*, *bfp*, *stx*, *ipaH*, and *elt/est*, to rule out EPEC, EHEC, EIEC, and ETEC.

### 2.6 Phylogroups and phylogeny

To determine the O:H serotypes of the E. coli isolates, *in silico* typing was performed using SerotypeFinder 2.0 (Joensen et al., 2015), with a selected threshold of 90% identity and 60% total serotype gene length. For phylogenetic analysis, single nucleotide polymorphisms (SNPs) were identified using CSI Phylogeny 4.1 (Centre for Genomic Epidemiology) with the *E. coli* reference sequence NZ_CP028166.1. SNP calling parameters included a minimum depth of coverage ≥ 10 × , SNP quality score ≥ 30, mapping quality score ≥ 25, and Z score ≥ 1.96 (Kaas et al., 2014). The resulting SNP data were used to construct phylogenetic trees, which were visualized and annotated using iTOL version 6 (Interactive Tree of Life).

### 2.7 Data collection and analysis

Statistical analysis was conducted using STATA software (StataCorp, 2019) on data organized in Microsoft Excel. Descriptive statistics, including frequencies and proportions, were used to summarize the phenotypic and genotypic characteristics of the *E. coli* isolates. To assess the relationship between phylogroups and sample types, Fisher’s exact test was performed using the Mehta-Patel network algorithm, which is robust for analyzing associations in sparse contingency tables with small, expected cell counts. Differences in the number of virulence genes across phylogroups were evaluated using the non-parametric Kruskal-Wallis test, while variations in resistance percentages for 12 antimicrobials across eight phylogroups were analyzed using Fisher’s exact test. Where applicable, *p*-values were calculated to determine the statistical significance of observed associations. Statistical significance was set at a threshold of *p* < 0.05.

### 2.8 Ethics approval and consent to participate

This study was approved by the Ethics Review Board of the National Hospital Abuja (NHA) under approval number NHA/EC/033/2018. To ensure the protection of patient privacy and confidentiality. A two-stage data encryption process was implemented to ensure that patient information remained strictly limited to the principal investigator and authorized research personnel. All data was de-identified and securely stored in compliance with ethical guidelines.

The bacterial isolates analyzed in this study were obtained from anonymized clinical specimens routinely submitted to the NHA laboratory for diagnostic purposes. All procedures adhered to the ethical guidelines and regulations established by the Ethics Review Board, ensuring the protection of patient rights and confidentiality throughout the research process.

## 3 Results

### 3.1 Characteristics of *Escherichia coli* isolates

A total of 107 *E. coli* isolates were recovered from clinical specimens obtained from 107 patients. The isolates were predominantly sourced from urine samples (68%), followed by blood (22%) and wound swabs (10%). The patients spanned a broad age range, from 2 months to 84 years, with a mean age of 45 years.

**FIGURE 1:**
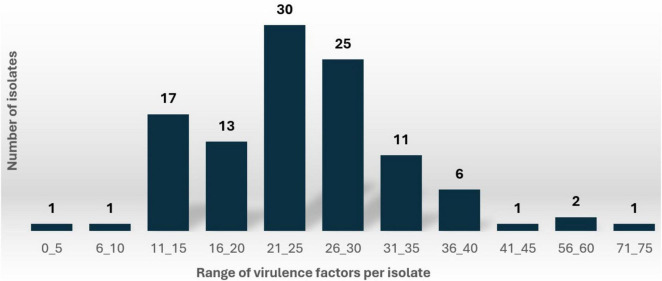
Distribution of isolates by number of virulence per *E. coli* isolate.

Comprehensive analysis identified 2,639 virulence genes across the isolates (Supplementary Table 1). When variants of the same gene were grouped (e.g., *yehA, yehB, yehC, yehD* grouped as *yeh*) the total number of distinct virulence genes was 2,021 (Supplementary Table 2). The number of virulence genes per isolate varied significantly, ranging from 7 to 48 (grouped format) and 7 to 71 (ungrouped format). As illustrated in Figure 1, the distribution of virulence factors (ungrouped format) revealed that 28% of isolates carried 21-25 virulence genes, followed closely by 23% of isolates (25 isolates) with 26-30 virulence genes.

### 3.2 Functional categorization of virulence genes

The 109 distinct virulence genes identified were categorized into nine functional groups (Table 1). The majority of virulence factors were associated with immune evasion and modulation (1,573 genes), followed by adherence, biofilm formation, and colonization (1,380 genes), and cellular invasion and intracellular survival (821 genes). Notably, some virulence factors, such as the *yeh* gene complex, exhibited multifunctional properties, contributing to adherence, biofilm formation, and colonization; invasion and intracellular survival; and immune evasion and modulation.

**TABLE 1:** Types of Virulence factors detected in *E. coli* isolates from clinical samples.

Category	Function	*n*	Virulence factor
1	Immune evasion and modulation	1,573	*aaiC, aap, aatA, aar, aggR, afaB, afaC, afaD, afaE, air, anr, AslA, cib, clbB, cnf1, cseA, fimH, focCsfaE, focG, EspI, hra, irp2, iss, lpfA, nlpI, ompT, ORF3, papA_F13, papA_F14, papA_F40, papA_F43, papA_F48, papA_F7-2, papA_fsiA_F16, papC, pic, sat, sepA, sigA, senB, shiA, shiB, tcpC, tia, traJ, traT, vat, yehA, yehB, yehC, yehD, yfcV*
2	Adherence, biofilm, and colonization	1,380	*aaiC, aap, aatA, aar, aamR, aggR, afaB, afaC, afaD, afaE, agg3C, agg3D, agg4A, agg4C, agg4D, agg5A, air, anr, AslA, C719-09_SefB:unpublished, cseA, csgA, dhaK, eilA, fdeC, fimH, focCsfaE, focG, hra, iha, lpfA, nlpI, mrkA, ompT, papA_F13, papA_F14, papA_F40, papA_F43, papA_F48, papA_F7-2, papA_fsiA_F16, papC, pic, sfaD, sfaE, sfaS, tia, vat, yehA, yehB, yehC, yehD, yfcV*
3	Invasion and intracellular survival	821	*aaiC, AslA, cnf1, espY2, fimH, focCsfaE, focG, EspI, ibeA, nlpI, papC, pic, senB, tia, vat, yehA,yehB, yehC, yehD, yfcV*
4	Iron acquisition, uptake, transport, and storage	452	*chuA, tibC, dhaK, fyuA, iha, iroN, ireA, irp2, iucC, iutA, sitA*
5	Toxin production and secretion	353	*aatA, astA, cea, cia, cma, colE5, colE6, cib, clbB, clpK1, cnf1, cvaC, fimH, focCsfaE, focG, EspI, hlyA, hlyE, hlyF, iha, mcmA, pic, sat, sepA, sigA, senB, terC, vat*
6	Stress response and regulation	344	*aar, aggR. afaA. Anr, clpK1, dhaK, eilA, fimH, focCsfaE, focG, hra, nlpI, ORF3*
7	Cell wall and membrane biogenesis	216	*afaC, agg3D, agg4C, agg4D, csgA, kpsE, kpsMII, kpsMII_K1, kpsMII_K4, kpsMII_K5, kpsMII_K52, kpsMII_K6, kpsMII_K7, mchB, neuC, ORF3, ORF4*
8	Nutrient acquisition and metabolism	74	*cea, cia, cma, colE5, colE6, dhaK, mchB, mchC, mchF, ompT, ORF3, ORF4*
9	Motility and Chemotaxis	4	*Aap*

### 3.3 Frequency and types of virulence factors detected in *Escherichia coli* isolates

The 107 *E. coli* isolates analyzed in this study displayed a diverse range of virulence genes with a total of 2,021 virulence genes identified or 2,639 when including gene variants separately. The most prevalent was the *yeh* gene complex, which contributes to adherence, invasion, and immune evasion, and was detected in all isolates (100%). Specific variants of the *yeh* complex were present at high frequencies, including *yehC* (97.2%), *yehD* (96.3%), *yehB* (93.5%), and *yehA* (87.9%). The complete distribution of gene alleles is detailed in Supplementary Table 1, with an abridged version provided in Table 2.

**TABLE 2:** Prevalence of virulence genes in 107 *E. coli* isolates.

Gene	N (%)	Gene	N (%)	Gene	N (%)
	107 (100.0)	*kps*	48 (44.9)	*cvaC*	7 (6.5)
*terC*	103 (96.3)	*Gad*	45 (42.1)	*Pic*	7 (6.5)
*nlpI*	101 (94.4)	*ompT*	44 (41.1)	*tcpC*	7 (6.5)
*cseA/csgA*	100 (93.5)	*sat/sepA/sigA*	39 (36.4)	*dhaK*	6 (5.6)
*fdeC*	98 (91.6)	*iha*	37 (34.6)	*ibeA*	6 (5.6)
*fimH*	92 (86.0)	*shiA/B*	33 (30.8)	*vat*	6 (5.6)
*hly*	85 (79.4)	*usp*	33 (30.8)	*agg*	4 (3.7)
*hha*	77 (72.0)	*hra*	30 (28.0)	*neuC*	4 (3.7)
*ireA/iroN/irp2*	71 (66.4)	*cea/cia/cib/clbB/cma/col*	26 (24.3)	*sfa*	4 (3.7)
*traJ/T*	70 (65.4)	*tia*	26 (24.3)	*focG/focCsfaE/G*	3 (2.8)
*fyuA*	68 (63.6)	*eilA*	20 (18.7)	*nfaE*	3 (2.8)
*iucC/iutA*	67 (62.6)	*senB*	20 (18.7)	*tibC*	3 (2.8)
*Anr*	65 (60.7)	*air*	19 (17.8)	*aaiC*	2 (1.9)
*AslA*	64 (59.8)	*espI/espY2*	18 (16.8)	*SefB*	2 (1.9)
*sitA*	64 (59.8)	*aap/aamR/aar/aatA*	17 (15.9)	*clpK1*	2 (1.9)
*Iss*	60 (56.1)	*afa*	16 (15.0)	*ORF3/4*	2 (1.9)
*lpfA*	54 (50.5)	*astA*	15 (14.0)	*mchB/C/F*	1 (0.9)
*papA*	50 (46.7)	*capU*	10 (9.3)	*mcmA*	1 (0.9)
*chuA*	48 (44.9)	*cnf1*	10 (9.3)	*mrkA*	1 (0.9)

Other frequently detected virulence factors were *nlpI* (immune evasion, adherence, intracellular survival, and stress response, 94.4%), *cseA/csgA* (immune evasion, adherence, cell wall and membrane biogenesis, 93.5%), *terC* (toxin production and secretion, 96.3%), and *fdeC* (adherence, biofilm, and colonization, 91.6%). Notably, the *fimH* gene, associated with our functional categories 1, 2, 3, 5, and 7, was present in 86.0% of the isolates. Additionally, iron acquisition-related genes from functional category 4, such as *fyuA* (62.6%), *irp2* (59.8%), *iucC* (62.6%), and *iutA* (60.7%) were prominent among the virulence factors.

### 3.4 Distribution and role of virulence genes with mobile genetic elements (MGEs)

**FIGURE 2:**
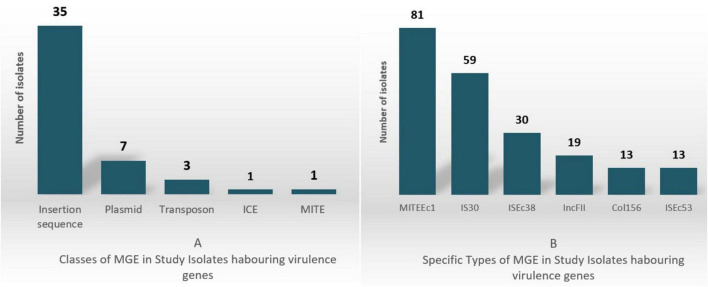
MGEs and virulence gene associations. **(A)** MGE classes; **(B)** Specific MGE types.

In the 107 *E. coli* isolates analyzed, virulence genes were found located on 48 unique MGEs, including Integrative Conjugative Elements (ICE), miniature inverted-repeat transposable elements (MITEs), insertion sequences, transposons, and plasmid incompatibility types. The classes and most prevalent MGE types are shown in Figure 2 with 2A depicting the classes and 2B depicting the specific classes.

MITEEc1 and IS30 were the most common, detected in 81 and 59 isolates, respectively. MITEEc1 frequently carried the immune evasion gene *terC* (46/55 instances), while IS30 was found alongside the SPATE genes *sat/sepA/sigA* (15/20 instances). ISEc38, identified in 30 isolates, often contained genes like *fyuA* (13 instances) and *irp2* (11 instances), which are involved in iron acquisition and bacterial survival.

Virulence genes involved in immune evasion and intracellular survival (e.g., *yeh*, *yehB*, *nlpI*) were more frequently located on MGEs compared to adherence-related genes like *fimH*, which were predominantly found on the chromosome. This distribution suggests a potential selective advantage for immune evasion genes in mobile forms, facilitating bacterial survival in host environments.

A total of 27 IncF-type plasmids were identified as carriers of virulence genes. Among these, IncFII plasmids were the most prevalent, found in 19 isolates, with the conjugative transfer genes *traJ/traT* present in 16 of these. Additionally, Col156 plasmids were detected in 13 isolates, all of which carried the *senB* gene, a key factor in immune evasion and cellular invasion. Supplementary Table 3 shows in more detail the virulence genes along MGE association. Entries indicate presence (1) or absence (0) of MGE linkage, and MGE type (e.g., ISEc38, ICKp1) if identified.

**FIGURE 3:**
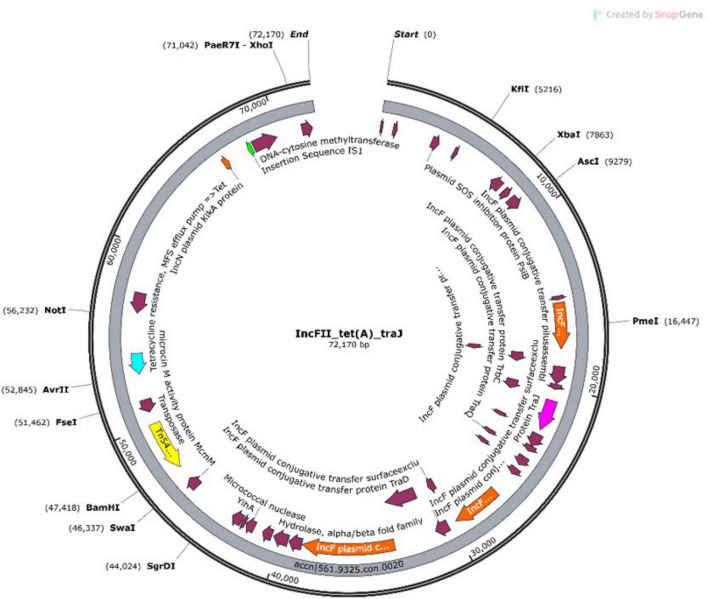
Annotation for NHA013 *IncFLL* plasmid.

Some plasmids simultaneously harbored both AMR genes and virulence genes, emphasizing their dual role in pathogenicity and resistance dissemination. Some of which include NHA068 with IncFIC plasmid having both the betalactam resistance gene *blaTEM-1B* and the virulence gene *traT*. A representative example from NHA013 shown in the plasmid annotation (Figure 3).

The annotated map highlights an IncFII plasmid carrying a transposon (Tn5403), virulence genes (*traJ/traT*), and the resistance gene *tet(A)*. These features were identified on contigs 19 and 20. The detailed annotation of contig 20 provides insight into the genetic architecture of the plasmid and its potential role in mediating multidrug resistance and virulence.

#### 3.4.1 Multi-gene clusters on MGEs

Some insertion sequences, such as ISSpu2 and IS5, hosted clusters of virulence genes within single isolates. For example, isolate NHA101 carried ISSpu2 with the genes *aatA*, *agg4A*, *aggR*, and *ORF3*, as well as an IS5 insertion sequence containing eight virulence genes: *ORF4*, *aatA*, *agg4A*, *afaD*, *aap*, *agg4C*, *agg4D*, and *ORF3*.

### 3.5 Correlation analysis of STs, virulence genes, AMR genes, MAR index, and comparative analysis of major vs. minor clones with DRQR mutations

#### 3.5.1 AMR gene landscape and MAR index

As we previously reported for this dataset (Medugu et al., 2022), 57 distinct AMR genes were found for strains in this study (Supplementary Table 4). The predominant AMR genes detected were β-lactam resistance genes with 13 different variants including the classical ESBL *bla*_CTX–M_ type (7 types), and *AmpC* producing *bla*_CMY_ type (1). Other β-lactam genes found included *bla*_OXA_ (2) and *bla*_TEM_ (2). Also reported were *bla*_NDM–1_ (1) and *bla*_NDM–5_ (4). There were ten genes detected for aminoglycoside resistance including *aac(3*′*)-IIa* and *aac*(3)*-Iie* found in 30% and 29% of isolates respectfully. We also reported genes conferring erythromycin, macrolide, phenicol, tetracycline, sulfonamide, and trimethoprim resistance (Supplementary Table 4). The multiple antibiotic resistance index (MAR index) ranged from 0.1 to 1 with a mean of 0.6 as detailed in Supplementary Table 4.

#### 3.5.2 Spearman’s correlation analysis

Spearman’s correlation analysis revealed significant associations between multidrug resistance and virulence determinants. A robust positive correlation was observed between the MAR Index and the Number of AMR genes (ρ = 0.813, *p* < 0.001), indicating that STs with higher multidrug resistance indices harbored greater AMR gene diversity. Similarly, the Number of virulence genes demonstrated a strong positive correlation with Virulence Variety (ρ = 0.741, *p* < 0.001), underscoring that STs with higher virulence gene counts also exhibited broader functional diversity in virulence factors. Moderate correlations were identified between AMR genes and Virulence Variety (ρ = 0.598, *p* = 0.006), suggesting a co-occurrence of antibiotic resistance and virulence diversification. The MAR Index exhibited a marginal, non-significant trend with Virulence Variety (ρ = 0.432, *p* = 0.057), hinting at a potential interplay between multidrug resistance and virulence complexity that warrants further investigation. No significant associations were detected between the MAR Index and Number of virulence genes (ρ = 0.221, *p* = 0.351) or between AMR genes and virulence gene counts (ρ = 0.128, *p* = 0.594).

#### 3.5.3 Major vs. minor clones and double QRDR mutations

Analysis of quinolone resistance-determining region (QRDR) mutations revealed a striking predominance of *gyrA_S83L* and *parC_S80I* double-serine mutations among high-risk clones. A total of 95.2% (40/42) of major (high-risk) clones harbored both mutations, compared to 68.9% (42/61) of minor (low risk) clones (Table 3; Supplementary Table 7). Notably, 21.3% (13/61) of minor clones lacked QRDR mutations entirely, while only 4.8% (2/42) of major clones exhibited this phenotype. Major clones demonstrated significantly higher antimicrobial resistance (MAR index: 0.7 vs. 0.5) and virulence gene carriage (24.2 vs. 22.3 genes/isolate) compared to minor clones. This near-universal presence of double-serine mutations in major clones may correlate with their enhanced capacity to co-maintain resistance and virulence determinants without apparent fitness trade-offs (*p* > 0.05).

**TABLE 3:** Virulence and resistance characteristics of major and minor *E. coli* sequence types and qRDR double-serine mutations.

Clone type	ST	MAR index (average)	Number of AMR genes (average)	No of virulence genes (average)	Virulence variety (number)	gyrA_S83L (average)	parC_S80I (average)	No. of gyrA_S83 and ParC_S80 mutations (sum)	Percentage of isolates with double mutations
Major	ST131 (23)	0.6	13.3	28.7	72.0	1.0	1.0	2.0	98%
Major	ST 410 (15)	0.7	14.6	21.1	64.0	0.9	1.0	1.9	97%
Major	ST 648 (7)	0.7	11.9	25.4	48.0	1.0	1.0	2.0	100%
Major	ST10 (2)	0.6	10.0	12.0	23.0	1.0	1.0	2.0	100%
Major	ST 69 (2)	0.4	10.0	25.5	34.0	0.5	0.5	1.0	50%
Major	ST 405 (2)	0.6	14.0	30.5	32.0	1.0	0.5	1.5	75%
Major	ST 617 (5)	0.8	17.6	21.8	41.0	1.0	1.0	2.0	100%
Major	ST167 (3)	0.5	11.5	17.5	33.0	1.0	1.0	2.0	100%
Major	ST 354 (2)	0.5	7.5	25.0	39.0	1.0	1.0	2.0	100%
Major	ST 101 (4)	1.0	21.3	22.8	91.0	1.0	1.0	2.0	100%
Minor	ST38 (2)	0.7	14.5	37.5	38.0	0.5	0.5	1.0	50%
Minor	ST 2006 (4)	0.6	12.5	20.0	20.0	1.0	1.0	2.0	100%
Minor	ST156 (3)	0.5	10.0	21.3	40.0	1.0	1.0	2.0	100%
Minor	ST 6332 (2)	0.6	14.0	17.0	17.0	1.0	1.0	2.0	100%
Minor	ST151 (2)	0.3	7.0	19.5	27.0	0.5	0.5	1.0	50%
Minor	ST 205 (2)	0.7	10.5	35.5	52.0	1.0	1.0	2.0	100%
Minor	ST 216 (2)	0.0	4.0	24.0	35.0	0.0	0.0	0.0	0%
Minor	ST 652 (2)	0.5	12.0	28.0	29.0	0.0	0.0	0.0	0%
Minor	ST 744 (2)	0.6	12.0	15.0	15.0	1.0	1.0	2.0	100%
Minor	ST 998 (2)	0.5	8.5	27.5	42.0	1.0	0.0	1.0	50%

STs represented by only a single isolate have been removed from this table; their details are provided in Supplementary Table 7.

A Fisher’s exact test revealed a statistically significant association between clone type and the Single/Double/Nil outcome (*p* < 0.0001). Data in Table 3 and further detailed in Supplementary Table 4. Specifically, among Major clones, the vast majority displayed double mutation (61/65, 94%), with very few exhibiting the Single (3/65, 5%) or Nil (1/65, 2%) phenotype. In contrast, Minor clones were significantly less likely to present double mutation (21/38, or 55%) and were more frequently associated with the Nil outcome (12/38, or 32%), with a small proportion showing the Single phenotype (5/38, or 13%). Double mutations were found in an average of 88% of major clones and 56% of minor clones and Fisher’s exact test for the presence and absence and absence of double mutations demonstrated a statistically significant association between clone type and the percentage of isolates with double mutations (*p* = 0.0258). Supplementary Table 7 details the correlations between the major and minor clones alongside AMR genes, Virulence genes, and specific QRDR mutations.

### 3.6 Pathotypes of Escherichia coli

All 107 *E. coli* isolates were subjected to *Insilco* pathotyping. Of these, 58 (54.2%) fulfilled the Johnson criteria for ExPEC. Within this “typical ExPEC” subset, 42 (72.4% of 58) also satisfied the Spurbeck/Hooton–Mobley scheme and were thus classified as UPEC; 13 (22.4%) were deemed ExPEC−only (i.e., meeting Johnson criteria but not the Spurbeck/HM definition); and 3 (5.17%) carried the EAEC regulator gene (*aggR*), making them ExPEC/EAEC hybrids.

A further 47 isolates (43.93%) were designated “atypical ExPEC” because they possessed various ExPEC−associated genes yet did not meet either the Johnson or the Spurbeck/HM thresholds. One isolate (0.93%) fulfilled only the Spurbeck/HM definition but did not have the required two Johnson markers, so it was labeled “UPEC−only.” Finally, one isolate (0.93%) could not be classified under any of the applied criteria and was reported as unclassified (Supplementary Table 6).

### 3.7 Distribution of phylogroups in *Escherichia coli* isolates originating from clinical samples

The distribution of *E. coli* phylogroups across different clinical sample types is summarized in Table 4. Among the 107 isolates analyzed, phylogroup B2 was the most prevalent, accounting for 32.7% (*n* = 35) of the isolates, followed by phylogroup B1 at 23.4% (*n* = 25). The least common phylogroup was G, observed in only 2.8% (*n* = 3) of the isolates.

**TABLE 4:** Distribution of *E. coli* phylogroups according to sample type.

Sample type	A (%)	B1 (%)	B2 (%)	C (%)	D (%)	E (%)	F (%)	G (%)	Total
Aspirates	1 (33.3)	2 (66.7)	0 (0.0)	0 (0.0)	0 (0.0)	0 (0.0)	0 (0.0)	0 (0.0)	3
Blood cultures	1 (4.6)	3 (13.6)	11 (50.0)	2 (9.1)	2 (9.1)	1 (4.6)	1 (4.6)	1 (4.6)	22
Cerebrospinal fluid (CSF)	0 (0.0)	0 (0.0)	2 (66.7)	1 (33.3)	0 (0.0)	0 (0.0)	0 (0.0)	0 (0.0)	3
Endocervical swabs	3 (50.0)	0 (0.0)	3 (50.0)	0 (0.0)	0 (0.0)	0 (0.0)	0 (0.0)	0 (0.0)	6
Stool samples	0 (0.0)	1 (50.0)	1 (50.0)	0 (0.0)	0 (0.0)	0 (0.0)	0 (0.0)	0 (0.0)	2
Urine samples	11 (18.0)	15 (24.6)	16 (26.2)	4 (6.6)	4 (6.6)	2 (3.3)	7 (11.5)	2 (3.3)	61
Wound swabs	0 (0.0)	4 (40.0)	2 (20.0)	0 (0.0)	0 (0.0)	0 (0.0)	4 (40.0)	0 (0.0)	10
Total	16 (15.0)	25 (23.4)	35 (32.7)	7 (6.5)	6 (5.6)	3 (2.8)	12 (11.2)	3 (2.8)	107

Fisher’s exact test (Mehta-Patel network algorithm): *p* = 0.572.

When stratified by sample type, phylogroup B2 was most frequently identified in cerebrospinal fluid (CSF) cultures (66.7%, 2/3) and blood cultures (50.0%, 11/22). In contrast, phylogroup A was predominant in endocervical swabs (50%, 3/6), while phylogroup F was most common in wound swabs (40%, 4/10). Urine samples exhibited a more diverse distribution, with phylogroups B2 (26.2%, 16/61) and B1 (24.6%, 15/61) being the most frequently observed.

Statistical analysis using the Chi-square test revealed no significant relationship between phylogroup and sample type (χ^2^ = 41.68, *p* = 0.485). This suggests that the distribution of *E. coli* phylogroups is independent of the clinical sample type.

### 3.8 Relationship between phylogroups and virulence factors

**FIGURE 4:**
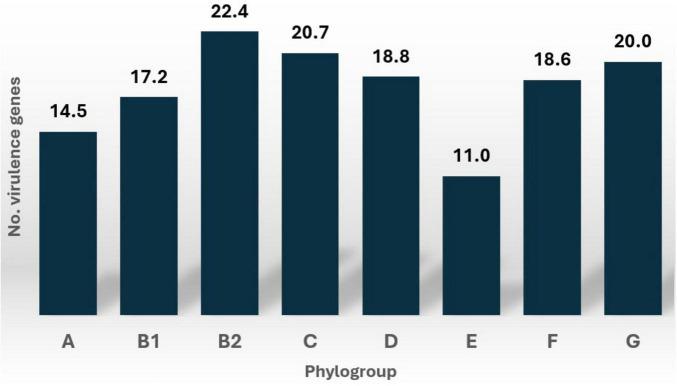
Distribution of virulence genes for each phylogroup across 107 *E. coli* strains.

The number of virulence genes varied across *E. coli* phylogroups, with phylogroup B2 exhibiting the highest median count. This is shown in detail on Supplementary Table 5. However, the Kruskal-Wallis test revealed no statistically significant differences in the number of virulence genes across phylogroups (H = 7.15, *p* = 0.413). This suggests that the distribution of virulence gene counts is consistent across the phylogroups analyzed in this study. Figure 4 illustrates the distribution of virulence gene counts for each phylogroup across the 107 *E. coli* strains.

Specific virulence factors, such as *chuA*, *yfcV*, *fimH*, and *cnf1*, were more frequently detected in phylogroup B2. In contrast, other virulence factors, including *sfaS*, *astA*, *csgA*, *fdeC*, *nlpI*, *terC*, and the *yeh* gene complex, were distributed across various phylogroups with minimal variations. Notably, phylogroups F and G were completely devoid of the *csgA* gene, which was prevalent in other phylogroups. Detailed information on the distribution of these virulence factors across phylogroups is provided in Supplementary Table 1.

### 3.9 Phylogroups and antimicrobial resistance

The AMR profiles of *E. coli* isolates varied significantly across phylogroups, as detailed in Table 5. Phylogroup B2 demonstrated high resistance rates to ampicillin (97.1%, *n* = 34), ceftriaxone (85.7%, *n* = 30), and sulfisoxazole (97.1%, *n* = 34), while phylogroup B1 showed complete resistance to ampicillin (100%, *n* = 25) and tetracycline (100%, *n* = 25), along with high resistance to trimethoprim/sulfamethoxazole (96.0%, *n* = 24).

**TABLE 5:** Antimicrobial resistance patterns among the phylogroups in *E. coli* isolates from clinical samples.

Antimicrobial	A	B1	B2	C	D	E	F	G
	*n* = 16	*n* = 25	*n* = 35	*n* = 7	*n* = 6	*n* = 3	*n* = 12	*n* = 3
Amoxicillin/clavulanic Acid	3 (18.75)	8 (32.0)	12 (34.3)	2 (28.6)	1 (16.7)	2 (66.7)	3 (25.0)	0 (0.0)
Ampicillin	12 (75.0)	25 (100.0)	34 (97.1)	7 (100.0)	5 (83.3)	3 (100.0)	12 (100.0)	3 (100.0)
Cefoxitin	2 (12.5)	6 (24.0)	3 (8.7)	1 (14.3)	1 (16.7)	2 (66.7)	3 (25.0)	0 (0.0)
Ceftriaxone	10 (62.5)	21 (84.0)	30 (85.7)	6 (85.7)	5 (83.3)	2 (66.7)	9 (75.0)	3 (100.0)
Chloramphenicol	5 (31.25)	10 (40.0)	5 (14.3)	1 (14.3)	2 (33.3)	1 (33.3)	4 (33.3)	1 (33.3)
Gentamicin	4 (25.0)	16 (64.0)	19 (54.3)	5 (71.4)	4 (66.7)	2 (66.7)	8 (66.7)	2 (66.7)
Tetracycline	13 (81.25)	25 (100.0)	32 (91.4)	7 (100.0)	6 (100.0)	3 (100.0)	10 (83.3)	3 (100.0)
Trimethoprim/sulfamethoxazole	12 (75.0)	24 (96.0)	33 (94.3)	5 (71.4)	5 (83.3)	3 (100.0)	2 (16.7)	3(100.0)
Sulfisoxazole	12 (75.0)	23 (92.0)	34 (97.1)	6 (85.7)	5 (83.3)	3 (100.0)	10 (83.3)	3 (100.0)
Ciprofloxacin	11 (68.75)	22 (88.0)	29 (82.9)	7 (100.0)	6 (100.0)	2 (66.7)	11 (91.7)	2 (66.7)
Nalidixic Acid	13 (81.25)	23 (92.0)	32 (91.4)	7 (100.0)	6(100.0)	3 (100.0)	12 (100.0)	2 (66.7)
Meropenem	0 (0.0)	3 (12.0)	4 (11.4)	1 (14.3)	0 (0.0)	0 (0.0)	0 (0.0)	0 (0.0)

Fisher’s exact test (Mehta-Patel algorithm): *p* = 0.225.

Resistance to amoxicillin/clavulanic acid was most prevalent in phylogroup E (66.7%, *n* = 2), whereas cefoxitin resistance was highest in phylogroup E (66.7%, *n* = 2) and phylogroup F (25.0%, *n* = 3). Notably, phylogroup G exhibited complete resistance to ampicillin, ceftriaxone, tetracycline, and sulfisoxazole, but no resistance to meropenem.

Resistance to meropenem was observed only in phylogroups B1 (12.0%, *n* = 3), B2 (11.4%, *n* = 4), and C (14.3%, *n* = 1). Resistance to ciprofloxacin and nalidixic acid was widespread, with phylogroup C showing 100% resistance to both antibiotics.

Kruskal-Wallis analysis of resistance percentages across 12 antibiotics indicated no significant difference among the 8 phylogroups [H (7) ≈ 10.52, *p* = 0.1625] and Fisher’s exact test (Mehta-Patel algorithm): *p* = 0.225 (Table 4).

### 3.10 Hierarchical clustering of virulence genes, plasmid association, and mobile genetic elements

A single nucleotide polymorphism (SNP)-based phylogenetic tree was constructed to examine the genetic relationships among the 107 *E. coli* isolates. It revealed distinct clustering of isolates according to their phylogroups, with B2 being the most genetically diverse. The presence of virulence genes, sequence types, plasmid associations, and MGEs was mapped onto the tree, illustrating their distribution across different phylogenetic lineages. Notably, virulence genes associated with immune evasion, adherence, and biofilm formation were more prevalent in phylogroup B2, whereas plasmid-encoded virulence genes were commonly found in isolates from multiple phylogroups.

**FIGURE 5:**
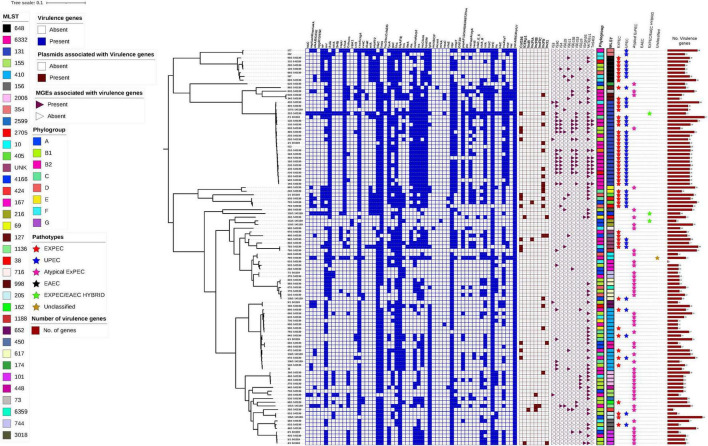
Hierarchical clustering of 107 Escherichia coli isolates based on the presence of virulence genes, plasmid association, and mobile genetic elements (MGEs). The heatmap displays virulence gene presence (blue) and absence (white), while plasmid association with virulence genes is indicated in dark red. MGEs associated with virulence genes are marked with pink triangles (present) or white triangles (absent). The color strips represent Phylogroups and MLSTs with each phylogroup coded in a distinct color. The Pathotypes are represented by different colored triangles. The SNP was based on the reference strain NZ_CP028166.1.

Figure 5 presents the SNP phylogenetic tree, alongside a heatmap indicating the presence (blue) or absence (white) of virulence genes. The association of virulence genes with plasmids is highlighted in dark red, while MGEs linked to virulence factors are represented by pink triangles. Sequence types are shown in a color strip. The bar chart on the right illustrates the number of virulence genes per isolate, and the phylogroups are color-coded.

## 4 Discussion

This study provides a comprehensive genomic analysis of MDR *E. coli* isolates from a tertiary hospital in Nigeria, offering insights into the relationships between virulence factors, phylogenetic diversity, and AMR profiles. Using WGS, we identified a complex genetic makeup with diverse phylogroups, widespread AMR, and over 100 distinct virulence genes spanning nine functional groups, many of which are located on MGEs. The finding of many of these genes on MGEs—including plasmids like IncFII and Col156—demonstrates how quickly these pathogens could gain and share virulence and resistance traits, potentially complicating clinical management. These findings underscore the urgent need for targeted interventions to curb the spread of MDR *E. coli* in resource-limited settings like Nigeria.

The identification of 2,021 virulence genes across nine functional categories highlights the pathogenic potential of these MDR-E isolates. The predominance of genes involved in immune evasion (1,573 genes), adherence/biofilm formation (1,380 genes), and iron acquisition (452 genes) aligns with the well-established mechanisms by which *E. coli* establishes and sustains infections (Croxen and Finlay, 2009; Kaper et al., 2004). The near-universal presence of the *yeh* gene complex (100%) and the high prevalence of *nlpI* (94.4%) and *fimH* (86%) align with previous findings in pathogenic *E. coli*, particularly ExPEC strains, where these genes are linked to enhanced host adaptability (Croxen and Finlay, 2009; Johnson and Russo, 2002; Kaper et al., 2004; Leimbach et al., 2013). The multifunctional nature of genes like *yeh* and *fimH* suggests they have evolved to thrive in diverse niches, from the urinary tract to the bloodstream and wound sites (Yürüyen et al., 2018). The *yeh* gene complex supports multifunctionality, contributing to adherence, immune evasion, and intracellular survival—traits essential for successful colonization and persistence in host environments (Bien et al., 2012; Ravan and Amandadi, 2015). Similarly, *fimH*, a type 1 fimbrial adhesin gene, plays a critical role in establishing adherence to host tissues and is also implicated in immune evasion, making it a potential candidate for therapeutic or vaccine-based interventions (Hojati et al., 2015).

Fimbriae play a pivotal role in *E. coli* pathogenicity by mediating adherence to host cells, a critical step for colonization, biofilm formation, and immune evasion. This study identified key fimbrial adhesins encoded by the *afa*, *sfa*, and *pap* gene clusters, which facilitate attachment to uroepithelial cells, sialic acid receptors, and kidney tissues, respectively, contributing to infections like UTIs (Bergsten et al., 2004; Boisen et al., 2008; Morschhäuser et al., 1993). Biofilm formation, supported by genes like *csgA*, *AslA*, and *neuC*, enhances resistance to antimicrobials and host defenses, with *FdeC* emerging as a potential vaccine target due to its role in adhesion and biofilm integrity (Vann et al., 2004). Iron acquisition systems, including *fyuA*, *irp2*, and *iutA*, allow *E. coli* to thrive in iron-limited environments, while immune-modulatory genes like *iss* and *hra* promote serum resistance and host tissue persistence, showing the bacterium’s adaptability and resilience in diverse host environments (Johnson et al., 2008; Promite and Saha, 2020).

A particularly striking finding is the finding of virulence genes with MGEs, such as MITEEc1 and IS30. This highlights the role of horizontal gene transfer in the dissemination of virulence determinants (Partridge et al., 2018). The frequent localization of immune evasion genes like *terC* on MGEs suggests that these genes benefit from being mobile, enabling their rapid spread among bacterial populations (Carattoli, 2013). This is especially concerning in hospital settings, where the exchange of MGEs could lead to the emergence of highly virulent and resistant strains (von Wintersdorff et al., 2025).

Plasmids, such as IncFII and Col156, were frequently found to carry both virulence and AMR genes. For example, IncFII plasmids, detected in 19 isolates, often harbored conjugative transfer genes (*traJ/traT*) in addition to AMR genes, potentially facilitating the rapid dissemination of these genetic elements (Carattoli, 2009). Col156 plasmids, identified in 13 isolates, consistently carried the *senB* gene, which is involved in immune evasion and cellular invasion (Smillie et al., 2010). The co-localization of virulence and resistance genes on the same MGEs underscores their potential dual role in driving the spread of both pathogenicity and resistance, posing a significant challenge for clinical management and infection control.

The presence of multi-gene clusters on MGEs, such as ISSpu2 and IS5, further emphasizes their role in the rapid evolution of pathogenic *E. coli* strains. For instance, isolate NHA101 carried ISSpu2 with genes like *aatA*, *agg4A*, and *aggR*, as well as an IS5 insertion sequence containing eight virulence genes, including *afaD* and *aap* (Dobrindt et al., 2004). These findings suggest that MGEs not only facilitate the spread of individual virulence genes but also enable the acquisition of entire virulence gene clusters, potentially leading to the emergence of hypervirulent strains.

Our sequence type (ST) analysis revealed that globally disseminated clones (ST131, ST410, ST648) exhibited greater diversity in virulence genes compared to minor clones (*p* = 0.028), aligning with prior reports linking these STs to heightened pathogenic potential in extraintestinal infections (Johnson and Stell, 2000; Pitout, 2012). For instance, ST131—a well-documented pandemic clone—carried a median of 15.7 virulence genes, including *fimH* (98%) and *fyuA* (89%), consistent with its association with urinary tract and bloodstream infections (Spurbeck et al., 2012).

A key observation in our study is the markedly high prevalence of QRDR double-serine mutations (gyrA_S83L and parC_S80I) in major clones. These mutations, demonstrated to confer a fitness advantage (Fuzi et al., 2020), may facilitate the acquisition and stable retention of a larger gene cargo without imposing significant fitness costs. In our dataset, nearly all major clones harbored these mutations, whereas a lower proportion was observed among minor clones. This near-universal presence of QRDR double-serine mutations in high-risk clones likely underpins their enhanced ability to co-maintain both multidrug resistance and a robust virulence repertoire. While previous studies have noted a potential virulence-resistance trade-off in ST131 (Shaik et al., 2017), our findings suggest that in high-transmission settings like Nigeria—where selective pressures such as antibiotic misuse are intense—the fitness advantage conferred by these QRDR mutations may offset any potential trade-offs. As a result, major clones can thrive even with the added burden of an extensive virulence gene repertoire. This provides a plausible explanation for our observation that MDR strains from major clones exhibit a more diverse virulence gene cargo compared to isolates from minor clones.

Three ExPEC/EAEC hybrids were identified, harboring canonical ExPEC virulence markers (*fyuA*, *chuA*, *kpsMTII*) alongside the enteroaggregative regulator *aggR*. These hybrids exemplify the genomic plasticity of *E. coli*, merging the iron acquisition systems and immune evasion strategies of ExPEC with the aggregative adherence and biofilm-forming capacity of EAEC (Lara et al., 2017; Sarowska et al., 2019). For instance, isolate NHA072 carried *aggR* alongside *papG* (P fimbriae) and *kpsMTII* (group 2 capsule synthesis), traits associated with urinary tract colonization and systemic invasion, respectively. Such hybrids are increasingly reported in clinical settings, where their dual virulence repertoires may facilitate colonization of both intestinal and extraintestinal niches, complicating diagnostic categorization and empiric therapy (Santos et al., 2020a). Their detection underscores the need for genomic surveillance to identify emerging pathotypes that evade conventional diagnostic frameworks.

While phenotypic AMR profiles for these isolates were previously published (Medugu et al., 2022), genomic analysis here underscores the role of MGEs in resistance dissemination. IncFII plasmids co-harbored *blaTEM-1B* and *traT* in 16 isolates, mirroring findings in other LMICs where such plasmids drive carbapenem and fluoroquinolone resistance (Carattoli, 2013). The strong correlation between AMR gene counts and resistance indices (ρ = + 0.85) highlights the predictive value of WGS for resistance surveillance. However, the persistence of meropenem resistance in phylogroups B1 (12%) and B2 (11%)—despite restricted carbapenem use in Nigeria—may suggest cryptic transmission via mobile elements or clonal expansion, warranting targeted screening.

Phylogroup B2 emerged as the most prevalent (32.7%) and clinically significant group, consistent with its well-documented association with ExPEC (Partridge et al., 2018). The higher diversity of virulence genes and AMR determinants in phylogroup B2 isolates underscores their potential to cause severe infections, such as sepsis, meningitis, and urinary tract infections (UTIs) (Johnson and Stell, 2000). The predominance of phylogroup B2 in cerebrospinal fluid (CSF) and blood cultures further emphasizes its role in invasive infections, necessitating heightened surveillance and targeted interventions (Pitout, 2012).

While the distribution of phylogroups across clinical sample types was not statistically significant, it still provides valuable epidemiological insights. For example, the predominance of phylogroup A in endocervical swabs and phylogroup F in wound swabs suggests niche-specific adaptations that warrant further investigation (Aguirre-Sánchez et al., 2022; Stoppe et al., 2017). The lack of a strong association between phylogroup and sample type, however, indicates that other factors—such as host immune status and antibiotic exposure—may play a more decisive role in infection outcomes (Aguirre-Sánchez et al., 2022; Stoppe et al., 2017; Tenaillon et al., 2010).

## 5 Limitations

While this study provides valuable insights into the genomic epidemiology of multidrug-resistant *Escherichia coli* (MDR-E) in Nigeria, several limitations must be acknowledged. The cross-sectional design of this research restricts our ability to establish causal relationships between specific genomic features and clinical outcomes. WGS offers extensive genomic data, this study did not incorporate functional assays to validate the roles of identified virulence genes and their interactions with host factors. Future research should integrate experimental approaches to elucidate the functional significance of these virulence determinants and their impact on infection severity and patient outcomes.

## 6 Conclusion

This study highlights the genomic diversity and complexity of multidrug-resistant *Escherichia coli* circulating in Nigeria, characterized by a wide range of sequence types, phylogroups, and virulence–resistance profiles, and a high prevalence of virulence genes frequently associated with MGEs. The high prevalence of ExPEC and UPEC pathotypes, alongside atypical ExPEC and ExPEC/EAEC hybrids, reflects the shifting landscape of *E. coli* pathogenicity, where classical pathotype boundaries are increasingly blurred. Notably, the universal presence of the yeh gene complex across all isolates highlights a critical virulence mechanism facilitating adherence, invasion, and immune evasion. The co-localization of virulence genes on MGEs, particularly plasmids such as IncFII and Col156, exemplifies the role these elements play in disseminating pathogenicity traits. This genetic linkage poses significant challenges for infection control and clinical management, as it could enable the rapid spread of highly virulent *E. coli* strains within and potentially beyond healthcare settings.

These findings underscore the urgent need for enhanced genomic surveillance through routine WGS-based monitoring to enable early detection of virulent *E. coli* strains and timely interventions. Strengthening infection control measures, including strict hygiene and isolation protocols, is crucial to limiting MDR-E transmission in healthcare settings. Additionally, leveraging key virulence factors like the *yeh* gene complex and *fimH* for vaccine or antivirulence therapy development could provide novel treatment and prevention strategies. Finally, implementing national policies on AMR, regulating antibiotic sales, and promoting public education on responsible antibiotic use are essential to curbing the spread of MDR-E.

Addressing the spread of highly virulent and adaptable *E. coli* strains through these measures is imperative for improving patient outcomes and safeguarding public health in resource-limited settings like Nigeria.

## Data Availability

The datasets presented in this study can be found in online repositories. The names of the repository/repositories and accession number(s) can be found in the article/Supplementary material.

## References

[B1] Aguirre-Sánchez J. Valdez-Torres J. Del Campo N. Martínez-Urtaza J. Del Campo N. Lee B. (2022). Phylogenetic group and virulence profile classification in *Escherichia coli* from distinct isolation sources in Mexico. *Infect. Genet. Evol.* 106:105380. 10.1016/j.meegid.2022.105380 36283634

[B2] Aiken A. Rehman A. de Kraker M. Madrid L. Kebede M. Labi A. (2023). Mortality associated with third-generation cephalosporin resistance in Enterobacterales bloodstream infections at eight sub-Saharan African hospitals (MBIRA): A prospective cohort study. *Lancet Infect. Dis.* 23 1280–1290. 10.1016/S1473-3099(23)00233-5 37454672 PMC7617135

[B3] Bankevich A. Nurk S. Antipov D. Gurevich A. Dvorkin M. Kulikov A. (2012). SPAdes: A new genome assembly algorithm and its applications to single-cell sequencing. *J. Comput. Biol.* 19 455–477. 10.1089/cmb.2012.0021 22506599 PMC3342519

[B4] Baquero F. Tedim A. Coque T. (2013). Antibiotic resistance shaping multi-level population biology of bacteria. *Front. Microbiol.* 4:15. 10.3389/fmicb.2013.00015 23508522 PMC3589745

[B5] Bergsten G. Samuelsson M. Wullt B. Leijonhufvud I. Fischer H. Svanborg C. (2004). PapG-dependent adherence breaks mucosal inertia and triggers the innate host response. *J. Infect. Dis.* 189 1734–1742. 10.1086/383278 15116313

[B6] Bien J. Sokolova O. Bozko P. (2012). Role of uropathogenic *Escherichia coli* virulence factors in development of urinary tract infection and kidney damage. *Int. J. Nephrol.* 2012:681473. 10.1155/2012/681473 22506110 PMC3312279

[B7] Boisen N. Struve C. Scheutz F. Krogfelt K. Nataro J. (2008). New adhesin of enteroaggregative *Escherichia coli* related to the Afa/Dr/AAF family. *Infect. Immun.* 76 3281–3292. 10.1128/IAI.01646-07 18443096 PMC2446688

[B8] Carattoli A. (2009). Resistance plasmid families in *Enterobacteriaceae*. *Antimicrob. Agents Chemother.* 53:2227. 10.1128/AAC.01707-08 19307361 PMC2687249

[B9] Carattoli A. (2013). Plasmids and the spread of resistance. *Int. J. Med. Microbiol.* 303 298–304. 10.1016/j.ijmm.2013.02.001 23499304

[B10] Carattoli A. Zankari E. García-Fernández A. Voldby Larsen M. Lund O. Villa L. (2014). In silico detection and typing of plasmids using PlasmidFinder and plasmid multilocus sequence typing. *Antimicrob. Agents Chemother.* 58 3895–3903. 10.1128/AAC.02412-14 24777092 PMC4068535

[B11] Clermont O. Dixit O. Vangchhia B. Condamine B. Dion S. Bridier-Nahmias A. (2019). Characterization and rapid identification of phylogroup G in *Escherichia coli*, a lineage with high virulence and antibiotic resistance potential. *Environ. Microbiol.* 21 3107–3117. 10.1111/1462-2920.14713 31188527

[B12] Clinical and Laboratory Standards Institute (0000). *CLSI M100*, 30th Edn. Wayne, PA: CLSI.

[B13] Croxen M. Finlay B. (2009). Molecular mechanisms of *Escherichia coli* pathogenicity. *Nat. Rev. Microbiol.* 8 26–38. 10.1038/nrmicro2265 19966814

[B14] Dobrindt U. Hochhut B. Hentschel U. Hacker J. (2004). Genomic islands in pathogenic and environmental microorganisms. *Nat. Rev. Microbiol.* 2 414–424. 10.1038/nrmicro884 15100694

[B15] Feldgarden M. Brover V. Gonzalez-Escalona N. Frye J. Haendiges J. Haft D. (2021). AMRFinderPlus and the reference gene catalog facilitate examination of the genomic links among antimicrobial resistance, stress response, and virulence. *Sci. Rep.* 11:12728. 10.1038/s41598-021-91456-0 34135355 PMC8208984

[B16] Fuzi M. Rodriguez Baño J. Toth A. (2020). Global evolution of pathogenic bacteria with extensive use of fluoroquinolone agents. *Front. Microbiol.* 11:504697. 10.3389/fmicb.2020.00271 32158437 PMC7052298

[B17] Gurevich A. Saveliev V. Vyahhi N. Tesler G. (2021). QUAST: Quality assessment tool for genome assemblies. *Bioinformatics* 29 1072–1075. 10.1093/bioinformatics/btt086 23422339 PMC3624806

[B18] Hojati Z. Zamanzad B. Hashemzadeh M. Molaie R. Gholipour A. (2015). The FimH gene in uropathogenic *Escherichia coli* strains isolated from patients with urinary tract infection. *Jundishapur. J. Microbiol.* 8:e17520. 10.5812/jjm.17520 25825648 PMC4376967

[B19] Iheanacho C. Eze U. (2022). Antimicrobial resistance in Nigeria: Challenges and charting the way forward. *Eur. J. Hosp. Pharm.* 29:119. 10.1136/ejhpharm-2021-002762 35190457 PMC8899635

[B20] Iregbu K. Medugu N. Abdullahi N. Aigbe A. Modibbo I. Nwajiobi-Princewill P. (2013). Urine culture contamination: A one-year retrospective study at the national hospital, Abuja. *Afr. J. Clin. Experimental Microbiol.* 14 101–104. 10.4314/ajcem.v14i2.10

[B21] Joensen K. Tetzschner A. Iguchi A. Aarestrup F. Scheutz F. (2015). Rapid and easy in silico serotyping of *Escherichia coli* isolates by use of whole-genome sequencing data. *J. Clin. Microbiol.* 53 2410–2426. 10.1128/JCM.00008-15 25972421 PMC4508402

[B22] Johansson M. Bortolaia V. Tansirichaiya S. Aarestrup F. Roberts A. Petersen T. (2021). Detection of mobile genetic elements associated with antibiotic resistance in *Salmonella enterica* using a newly developed web tool: Mobileelementfinder. *J. Antimicrob. Chemother.* 76 101–109. 10.1093/jac/dkaa390 33009809 PMC7729385

[B23] Johnson J. Russo T. (2002). Extraintestinal pathogenic *Escherichia coli*: “the other bad E coli”. *J. Lab. Clin. Med.* 139 155–162. 10.1067/mlc.2002.121550 11944026

[B24] Johnson J. Stell A. (2000). Extended virulence genotypes of *Escherichia coli* strains from patients with urosepsis in relation to phylogeny and host compromise. *J. Infect. Dis.* 181 261–272. 10.1086/315217 10608775

[B25] Johnson J. Murray A. Gajewski A. Sullivan M. Snippes P. Kuskowski M. (2003). Isolation and molecular characterization of nalidixic acid-resistant extraintestinal pathogenic *Escherichia coli* from retail chicken products. *Antimicrob. Agents Chemother.* 47 2161–2168. 10.1128/AAC.47.7.2161-2168.2003 12821463 PMC161843

[B26] Johnson T. Wannemuehler Y. Nolan L. (2008). Evolution of the iss gene in *Escherichia coli*. *Appl. Environ. Microbiol.* 74 2360–2369. 10.1128/AEM.02634-07 18281426 PMC2293169

[B27] Kaas R. Leekitcharoenphon P. Aarestrup F. Lund O. (2014). Solving the problem of comparing whole bacterial genomes across different sequencing platforms. *PLoS One* 9:e104984. 10.1371/journal.pone.0104984 25110940 PMC4128722

[B28] Kaper J. Nataro J. Mobley H. (2004). Pathogenic *Escherichia coli*. *Nat. Rev. Microbiol.* 2 123–140. 10.1038/nrmicro818 15040260

[B29] Lara F. Nery D. de Oliveira P. Araujo M. Carvalho F. Messias-Silva L. (2017). Virulence markers and phylogenetic analysis of *Escherichia coli* strains with hybrid EAEC/UPEC genotypes recovered from sporadic cases of extraintestinal infections. *Front. Microbiol.* 8:241651. 10.3389/fmicb.2017.00146 28217123 PMC5290387

[B30] Larsen M. Cosentino S. Rasmussen S. Friis C. Hasman H. Marvig R. (2012). Multilocus sequence typing of total-genome-sequenced bacteria. *J. Clin. Microbiol.* 50 1355–1361. 10.1128/JCM.06094-11 22238442 PMC3318499

[B31] Leimbach A. Hacker J. Dobrindt U. (2013). E. coli as an all-rounder: The thin line between commensalism and pathogenicity. *Curr. Top. Microbiol. Immunol.* 358 3–32. 10.1007/82_2012_303 23340801

[B32] Magiorakos A. Srinivasan A. Carey R. Carmeli Y. Falagas M. Giske C. (2012). Multidrug-resistant, extensively drug-resistant and pandrug-resistant bacteria: An international expert proposal for interim standard definitions for acquired resistance. *Clin. Microbiol. Infect.* 18 268–281. 10.1111/j.1469-0691.2011.03570.x 21793988

[B33] Manges A. Geum H. Guo A. Edens T. Fibke C. Pitout J. (2019). Global extraintestinal pathogenic *Escherichia coli* (ExPEC) lineages. *Clin. Microbiol. Rev.* 32:e00135-18. 10.1128/CMR.00135-18 31189557 PMC6589867

[B34] Medugu N. Aworh M. Iregbu K. Nwajiobi-Princewill P. Abdulraheem K. Hull D. (2022). Molecular characterization of multi drug resistant *Escherichia coli* isolates at a tertiary hospital in Abuja, Nigeria. *Sci. Rep.* 12:14822. 10.1038/s41598-022-19289-z 36050365 PMC9437016

[B35] Medugu N. Tickler I. Duru C. Egah R. James A. Odili V. (2023). Phenotypic and molecular characterization of beta-lactam resistant multidrug-resistant enterobacterales isolated from patients attending six hospitals in Northern Nigeria. *Sci. Rep.* 13:10306. 10.1038/s41598-023-37621-z 37365355 PMC10293160

[B36] Morales G. Abelson B. Reasoner S. Miller J. Earl A. Hadjifrangiskou M. (2023). The role of mobile genetic elements in virulence factor carriage from symptomatic and asymptomatic cases of *Escherichia coli* bacteriuria. *Microbiol. Spectr.* 11:e0471022. 10.1128/spectrum.04710-22 37195213 PMC10269530

[B37] Morschhäuser J. Uhlin B. Hacker J. (1993). Transcriptional analysis and regulation of the sfa determinant coding for S fimbriae of pathogenic *Escherichia coli* strains. *Mol. Gen. Genet.* 238 97–105. 10.1007/BF00279536 8097559

[B38] Nojoomi F. Ghasemian A. (2019). The relation of phylogroups, serogroups, virulence factors and resistance pattern of *Escherichia coli* isolated from children with septicemia. *New Microbes New Infect.* 29:100517. 10.1016/j.nmni.2019.100517 31080621 PMC6501060

[B39] Okeke I. Lamikanra A. Edelman R. (1999). Socioeconomic and behavioral factors leading to acquired bacterial resistance to antibiotics in developing countries. *Emerg. Infect. Dis.* 5 18–27. 10.3201/eid0501.990103 10081668 PMC2627681

[B40] Partridge S. Kwong S. Firth N. Jensen S. (2018). Mobile genetic elements associated with antimicrobial resistance. *Clin. Microbiol. Rev.* 31:e00088-e17. 10.1128/CMR.00088-17 30068738 PMC6148190

[B41] Partridge S. Kwong S. Firth N. Jensen S. (2024). Mobile genetic elements associated with antimicrobial resistance. *Clin. Microbiol. Rev.* 31:e00088-17. 10.1128/CMR.00088-17 30068738 PMC6148190

[B42] Pitout J. (2012). Extraintestinal pathogenic *Escherichia coli*: A combination of virulence with antibiotic resistance. *Front. Microbiol.* 20:9. 10.3389/fmicb.2012.00009 22294983 PMC3261549

[B43] Promite S. Saha S. (2020). *Escherichia coli* in respiratory tract infections: Evaluating antimicrobial resistance and prevalence of fimA, neuC and iutA virulence genes. *Gene Rep.* 18:100576. 10.1016/j.genrep.2019.100576

[B44] Ravan H. Amandadi M. (2015). Analysis of yeh fimbrial gene cluster in *Escherichia coli* O157:h7 in order to find a genetic marker for this serotype. *Curr. Microbiol.* 71 274–282. 10.1007/s00284-015-0842-6 26037379

[B45] Santos A. Santos F. Silva R. Gomes T. (2020a). Diversity of hybrid- and hetero-pathogenic *Escherichia coli* and their potential implication in more severe diseases. *Front. Cell. Infect. Microbiol.* 10:532570. 10.3389/fcimb.2020.00339 32766163 PMC7381148

[B46] Santos A. Silva R. Valiatti T. Santos F. Santos-Neto J. Cayô R. (2020b). Virulence potential of a multidrug-resistant *Escherichia coli* strain belonging to the emerging clonal group ST101-B1 isolated from bloodstream infection. *Microorganisms* 8:827. 10.3390/microorganisms8060827 32486334 PMC7355805

[B47] Sarowska J. Futoma-Koloch B. Jama-Kmiecik A. Frej-Madrzak M. Ksiazczyk M. Bugla-Ploskonska G. (2019). Virulence factors, prevalence and potential transmission of extraintestinal pathogenic *Escherichia coli* isolated from different sources: Recent reports. *Gut Pathog.* 11:10. 10.1186/s13099-019-0290-0 30828388 PMC6383261

[B48] Schmidt H. Hensel M. (2004). Pathogenicity islands in bacterial pathogenesis. *Clin. Microbiol. Rev.* 17 14–56. 10.1128/CMR.17.1.14-56.2004 14726454 PMC321463

[B49] Shaik S. Ranjan A. Tiwari S. Hussain A. Nandanwar N. Kumar N. (2017). Comparative genomic analysis of globally dominant ST131 clone with other epidemiologically successful extraintestinal pathogenic *Escherichia coli* (ExPEC) Lineages. *mBio* 8:e01596-17. 10.1128/mBio.01596-17 29066550 PMC5654935

[B50] Smillie C. Garcillán-Barcia M. Francia M. Rocha E. de la Cruz F. (2010). Mobility of plasmids. *Microbiol. Mol. Biol. Rev.* 74 434–452. 10.1128/MMBR.00020-10 20805406 PMC2937521

[B51] Sora V. Meroni G. Martino P. Soggiu A. Bonizzi L. Zecconi A. (2021). Extraintestinal pathogenic *Escherichia coli*: Virulence factors and antibiotic resistance. *Pathogens* 10:1355. 10.3390/pathogens10111355 34832511 PMC8618662

[B52] Spurbeck R. Dinh P. Walk S. Stapleton A. Hooton T. Nolan L. (2012). *Escherichia coli* isolates that carry vat, fyuA, chuA, and yfcV efficiently colonize the urinary tract. *Infect. Immun*. 80 4115–4122. 10.1128/IAI.00752-12 22966046 PMC3497434

[B53] Stoppe N. Silva J. Carlos C. Sato M. Saraiva A. Ottoboni L. (2017). Worldwide phylogenetic group patterns of *Escherichia coli* from commensal human and wastewater treatment plant isolates. *Front Microbiol.* 8:289063. 10.3389/fmicb.2017.02512 29312213 PMC5742620

[B54] Tenaillon O. Skurnik D. Picard B. Denamur E. (2010). The population genetics of commensal *Escherichia coli*. *Nat. Rev. Microbiol.* 8 207–217. 10.1038/nrmicro2298 20157339

[B55] Vann W. Daines D. Murkin A. Tanner M. Chaffin D. Rubens C. (2004). The NeuC protein of *Escherichia coli* K1 is a UDP N-acetylglucosamine 2-epimerase. *J. Bacteriol.* 186 706–712. 10.1128/JB.186.3.706-712.2004 14729696 PMC321479

[B56] von Wintersdorff C. Penders J. van Niekerk J. Mills N. Majumder S. van Alphen L. (2025). Dissemination of antimicrobial resistance in microbial ecosystems through horizontal gene transfer. *Front. Microbiol.* 7:174871. 10.3389/fmicb.2016.00173 26925045 PMC4759269

[B57] Wirth T. Falush D. Lan R. Colles F. Mensa P. Wieler L. (2006). Sex and virulence in *Escherichia coli*: An evolutionary perspective. *Mol. Microbiol.* 60 1136–1151. 10.1111/j.1365-2958.2006.05172.x 16689791 PMC1557465

[B58] World Health Organization (2022). *Global Antimicrobial Resistance and Use Surveillance System (GLASS).* Geneva: World Health Organization.

[B59] Yürüyen C. Daldaban Dinçer Ş Yanılmaz Ö Boz E. S. Aksaray S. (2018). [Surveillance of resistance in the intensive care units using a cumulative antibiogram]. *Mikrobiyol Bul.* 52 329–339. 10.5578/mb.67408 30522419

